# Migration and Mental Health in Mexico: Domestic Migrants, Return U.S. Migrants, and Non-Migrants

**DOI:** 10.3389/fpsyt.2019.00970

**Published:** 2020-02-03

**Authors:** Katharine M. Donato, Laura Caron, Erin Hamilton

**Affiliations:** ^1^ Walsh School of Foreign Service, Georgetown University, Washington, DC, United States; ^2^ University of California, Davis, Davis, CA, United States

**Keywords:** Mexican migration mental health depression anxiety internal migrants, migration mental health Mexico, depression anxiety Mexico internal migration, US migration from Mexico, mental health

## Abstract

In this paper, we use survey data from the Mexican Retrospective Demographic Survey (Encuesta Demográfica Retrospectiva) and National Survey of Households (Encuesta Nacional de Hogares) collected in 2017 to examine self-reports of depression, anxiety, chronic fatigue, and pain among domestic migrants, returned U.S. migrants, and non-migrants. Although self-reports do not always correspond to clinical diagnoses, they offer some insight into mental health, especially for those without a diagnosis because of limited access to services or stigma. Regression results reveal that domestic migrants, e.g., those who moved within Mexico, reported more anxiety, chronic fatigue, and pain, but risks for U.S. migrants were comparable to non-migrants, controlling for other characteristics. Findings from the decomposition analysis helps explain these findings. While domestic migrant vs. non-migrant differences result both from different migrant demographic attributes, such as age and gender, and differences in the effects of these characteristics between the groups, U.S. migrant vs. non-migrant differences in anxiety and pain emerge only after allowing for the relationship between each observed characteristic and the mental health outcome to vary. Thus, compared to domestic migrants, U.S. migrants are selected on characteristics associated with good mental health—they are positively selected—but those characteristics are not protective for them.

## Introduction

After more than 150 years of large-scale Mexico-U.S. migration, the first decade of the twenty-first century witnessed dramatic changes in migration (1). Sustained declines in Mexican fertility, growing investments in education throughout Mexico, and the U.S. economic downturn in 2008 led to net zero migration between the two countries (2) with the lowest flows of Mexican immigrants heading northward since the 1990s (3). Concomitantly, the U.S. economic recession and expanded border enforcement are associated with more deportations and voluntary return among migrants and their relatives (4, 5). In 2015 alone, approximately 500,000 U.S. born minors were living in Mexico (6). Their large presence, together with growing numbers of migrants from Guatemala, Honduras and El Salvador seeking asylum (7), led to growth in Mexico's foreign-born population. Between 2000 and 2015, it rose from approximately 538,000 to 1.2 million persons (8).

These extraordinary changes in the direction and composition of migration flows between Mexico and the United States offer a new opportunity to examine the mental health consequences of migration in Mexico. In this paper, we seek to gain insights about migration and mental health in Mexico during this new age of migration by asking two questions. First, does migration correlate with mental health symptoms during this new age of migration, and if so, how? Second, how much do migrant differences in mental health symptoms reflect selectivity, such that migrants are different from non-migrants before they migrate? To answer these questions, we use measures of depression, anxiety, chronic fatigue, and pain from nationally representative household survey data collected in Mexico in 2017. We examine variation in self-reported symptoms of depression, anxiety, chronic fatigue, and pain by migrant experience, net of relevant controls, and use decomposition methods to assess how differences in group characteristics correlate with observed differences in mental health symptoms.

## Literature Review

Mental health issues are a growing concern in Mexico. Medina-Mora et al. (9) found that 12.1% of the Mexican population in 2001-02 reported a psychiatric disorder in the last 12 months, although the prevalence of severe disorders was much lower (3.5%). Approximately one-quarter (26.1%) of Mexicans reported having at least one disorder in their lifetime, and the age at onset of a disorder was early -- with half of those reporting a disorder doing so by age 21 (10).

The consequences of psychiatric disorders are serious and, perhaps, growing in scope. For example, in both 2007 and 2017, depressive disorders, back pain, and anxiety disorders ranked among the top 10 problems causing the most disability in Mexico (11). Moreover, as Gonzalez and Alvarez (12) note, declines in mortality related to mental health problems have recently shifted. Despite a 29% decline in the number of deaths due to mental and behavioral disorders between 1998 and 2008, deaths related to these disorders rose by 33% between 2008 and 2014. The authors speculate that the U.S. economic crisis and Mexico's war on drugs, which began in 2006, underlie this recent upward trend.

Most early studies about Mexican migrant mental health relied on data about populations either in Mexico or in the United States.^1^ For example, using data collected from adults living in Mexican cities in 2001 and 2002, Borges et al. (18) examined variation in addictive behaviors related to alcohol and drugs. They found that those with prior U.S. migration experience, and non-migrants whose relatives were U.S. migrants, were more likely to have used alcohol, marijuana, or cocaine at least once, develop a substance use disorder, and have a current disorder, compared to other Mexicans. Using data collected in three Mexican border cities in 2005, Borges et al. (19) reported that both return migrants and non-migrants with migrant relatives had higher prevalence of alcohol and drug use, as well as substance use disorders, than other Mexicans.

Other early studies relied only on data collected in the United States, reporting mixed results. Alegria et al. (20) found that Mexican immigrants had lower risks of depressive and anxiety disorders than U.S. born Mexicans, and Pena et al. (21) reported lower rates of alcohol and drug use among Mexican immigrants compared to Mexicans born in the United States. By contrast, Gerst et al. (22) found that Mexican-born men and women aged 75 years and older had higher risks of more depressive symptoms compared to U.S. born Mexicans. However, over time, many studies suggest that as Mexicans spent more time in the United States, they experienced worse mental health (23–25). Mexican immigrants' psychological well-being is also undermined because they experience discrimination and are subject to racialized policies of exclusion (23–26).^2^

Building on existing studies of U.S. immigrants, Perreria and colleagues (28, 29) collected longitudinal information from several hundred U.S. immigrants to disentangle pre- and post-migration characteristics and their effects on mental health outcomes. Ornelas and Perreira (30) reported that high poverty pre-migration, stressful migration experiences, racial neighborhood problems, and racial/ethnic discrimination were associated with the onset of depressive symptoms among Latino immigrant parents. Potochnick and Perreira (31) found that migration stressors (such as traumatic events, discrimination, and unauthorized status) increased the risk of depressive symptoms and anxiety, but with longer U.S. residence and more support from families and teachers, adolescents had lower risks of depressive symptoms and anxiety. In addition, Perreira and Ornelas (29) considered how particular pre- and post-migration characteristics were related to the risk of trauma and post-traumatic stress disorder among adolescents and their caregivers.

Other recent studies consider the relationship between migration and mental health using binational data that permit comparisons of Mexican immigrants in the United States with Mexicans in Mexico. For example, combining data from epidemiological surveys of adults in Mexico and the United States, Breslau et al. (32) reported higher risks for first onset of any depressive or anxiety disorder, for depressive disorders as a group, and for anxiety disorders as a group, among U.S. migrants aged 18–25 compared to risks for non-migrant family members of migrants in Mexico. Among those who were aged 26–35, the risk of having any depressive or anxiety disorder was higher. Using data from adults aged 18 to 44 in households in Mexico and the United States, Breslau et al. (33) reported that the risks of conduct disorder – especially non-aggressive symptoms – were lowest among non-migrant Mexicans, higher among children of Mexican born immigrants, and highest among Mexican-American children of U.S. born parents. Drawing from nationally representative surveys in the United States and Mexico, Borges et al. (34) found elevated risks of alcohol and drug use, and alcohol and drug use disorders, among return and current U.S. migrants. Swanson etal. (35) examine also reported higher risks of binge eating disorder among Mexican migrants in the United States compared to Mexicans with no migrant family members. Borges et al. (36) also describe how, compared to Mexican non-migrants, Mexican return migrants, Mexicans currently living in the United States, and second and third generation Mexican Americans with immigrant parents or grandparents, had higher prevalence of anxiety and greater number of symptoms than Mexican non-migrants. Using data from three Texas border metropolitan areas and their sister cities in Mexico, Borges et al. (37) found higher risks of drug use and alcohol use disorder for migrants compared to Mexican non-migrants.

Finally, two studies rely on data from the Mexican Migration Project (MMP), which collects information about respondents' health from representative samples of households in different Mexican origin communities beginning in 2007 up to present. Ullmann et al. (38) found more favorable early-life health among male Mexican migrant household heads who returned to Mexico, but compared to non-migrants, migrants also had higher prevalence of emotional/psychiatric disorders, smoking, obesity, and heart disease. Using MMP data from household heads and spouses between 2007 and 2016, Donato et al. (39) examined gender differences in the health of Mexican return migrants and non-migrants. They found women were more positively selected on height than male migrants, and a stronger positive association between migration and smoking among women than men.

Thus, prior studies suggest that important differences in the risks of mental illness and mental health symptoms among Mexican non-migrants, return migrants, and current U.S. migrants. Despite some mixed results from studies using data on Mexican immigrants in the United States, most studies based on binational data sources suggest higher risks among migrants compared to non-migrants. Yet, not well understood is the extent of such differences during this new era of Mexican migration, when return migration is growing and Mexico-U.S. migration is declining. Furthermore, to our knowledge no studies consider whether and how mental health symptoms vary for domestic migrants within Mexico vs. U.S. return migrants and non-migrants. Therefore, in this article, we examine the mental health profiles of non-migrants, domestic migrants, and returned U.S. migrants. We also assess the extent to which selectivity underlies non-migrant vs. migrant differences in mental health symptoms. By doing so, our analysis considers whether and how migration is associated with deleterious effects on mental health.

## Data And Methods

In this paper, we use data from the 2017 Retrospective Demographic Survey (Encuesta Demográfica Retrospectiva, or EDER) to study the relationship between mental health and migration for adults ages 20–54 in Mexico. Because the EDER is a life history supplement given to 20–54 year olds in the National Survey of Households (Encuesta Nacional de Hogares, or ENH) sample, we created an analytic sample for our analysis by combining the EDER migration questions with ENH questions about mental health symptoms recorded at the time of the survey.^3^

A key strength of these data is that they contain information about individuals' current situation and their life histories. The EDER life history questionnaire was administered to one randomly selected 20–54 year-old member in each sampled ENH household. Because data were collected only from those residing in Mexican households, our analytic sample contains three migrant groups: 1) domestic migrants who lived in a Mexican state different from their birth state at the time of the survey (N=9,441); 2) U.S. migrants who returned back to Mexico (N=1,680);^4^ and 3) non-migrants (N=14,088).

Although the ideal data set would contain detailed information on mental health disorders and well-being and include questions that capture clinical definitions of conditions such as depression and anxiety, the data we use derive from self-reports of related symptoms. We recognize there may be limitations of self-reports about mental health, but argue that they are minimized because the data derive only from Mexicans currently living in Mexico, who we would expect to be exposed to similar social and cultural forces. Thus, despite limitations, we use variables for self-reported depression, anxiety, fatigue and pain because we can link them with data about migrants' life histories – an advantage that most data sets do not offer. Thus, we treat true mental health status as an underlying, unobserved variable approximated by these questions, and caution readers to keep in mind that the self-reported measures we use are not clinical measures of mental health disorders or mental illness.

### Variable Measurement

In this analysis, the main independent variable of interest, migration, appears in two of the survey questions. With respect to the first question, individuals are considered migrants if they reported having migrated or changed household location for less than one year. The second question refers to the location of the household for a year or more: respondents are considered migrants if they report that their household location was in the United States (U.S. migration) or in a different state of Mexico than their birthplace (domestic migration within Mexico) in any year prior to the survey. Respondents can be domestic migrants, U.S. return migrants, both domestic and U.S. return migrants, or a non-migrant. Non-migrant is the reference category.

The dependent variables derive from self-reported feelings about depression, anxiety, chronic fatigue, and chronic pain.^5^ The first two, depression and anxiety, are measured through two questions that examine current (at the time of the survey) frequency of these experiences. From this information, we created binary dependent variables that capture whether the respondent feels depressed (or anxious) on a weekly or daily basis. Although chronic fatigue and pain are not considered mental health symptoms themselves, because they are often related to mental health conditions and because stigma and lack of awareness of mental health disorders may lead to underreporting of psychiatric disorders, we include these two dependent variables.^6^ Drawing from questions that assess whether both existed in the last three months, we recoded respondents as having chronic fatigue or pain if they report feeling pain or fatigue most days or every day.

Among the independent variables, we control for age (in years) and age squared because we expect that the association between age and mental health is curvilinear. We control for gender and expect that, relative to men, women will be more likely to report all four outcomes. Education will be negatively associated; higher levels of education will be related to lower prevalence of mental health conditions. Education is coded as a set of dummy variables equal to primary school, junior high, high school, college, or at least some graduate school, with the reference defined as those without any schooling. Being indigenous and being employed are coded as two binary variables (whereby 1 equals indigenous or employed, 0 not); we expect they will also be negatively related to mental health. To capture social support, we compared currently married and formerly married to never marry. We expect that being currently married will reduce the likelihoods of depression and anxiety, but being formerly married will increase them. Similarly, we measure whether respondents are living with other relatives as a binary variable, whereby 1 equals living with these relatives and 0 otherwise. We also control for the number of children residing in households and for household wealth. We calculated the latter from a principal components analysis of data corresponding to ownership of VCR or DVD players, washing machine, and microwaves. Approximately 15% of the sample owns all four of these.

### Analytic Strategy

We begin by presenting descriptive differences in the mental health outcomes of Mexicans who never migrated, those with U.S. migrant experience, and internal migrants within Mexico. We follow with findings from multivariate models that predict depression, anxiety, fatigue and pain, and examine the effects of migration controlling for relevant variables. However, because these models may not give a full picture of the relationship between migrants' socioeconomic and demographic characteristics and their propensity to develop the observed mental health symptoms, we also present findings from Oaxaca-Blinder decompositions. These findings permit us to assess whether and how differences in non-migrant vs. migrant mental health symptoms are due to the characteristics of these groups, suggesting selectivity, and/or due to differences in the associations of these characteristics with the dependent variables.

The decomposition, explained in detail in the Methodological Appendix, allows us to breakdown differences in mental health between migrants and non-migrants into three components. The first portion refers to differences in personal characteristics of the groups, known as endowments. For example, if migrants are more likely to be male than female and men are less likely than women to report depression, anxiety, pain, or fatigue, the difference in prevalence of males in each migrant group may appear as a difference in the mental health of migrants vs. non-migrants. The second portion of the difference between migrants and non-migrants is due to differences in the magnitudes of the relationships between group characteristics and each of the mental health symptoms; this is often referred to as differences in coefficients or returns to characteristics. For example, if the effect of being female on mental health is stronger for migrants than non-migrants, this would be a difference due to differences in coefficients. Finally, a portion of the migrant vs. non-migrant difference may be due to differences in the interactions between characteristics and coefficients. These are differences that occur when the total combined effect of differences in characteristics and coefficients is greater (or less) than the sum of the two parts. For example, if gender matters more for migrants and there are gender imbalances between the groups of migrants and non-migrants, these two effects together may combine to have an even larger effect.

Two additional technical points before we begin. First, although logistic regression models are usually used with 0,1 dependent variables, we estimate OLS models with standard errors that correct for the heteroscedasticity imposed by the linear models to facilitate use of the Oaxaca-Blinder decomposition method. In a separate analysis not presented here but available upon request, we also estimated logistic regression models and used the Fairlie decomposition, which partially adapts the Oaxaca-Blinder method to nonlinear models (40). After comparing these results to the OLS models and Oaxaca-Blinder decomposition, we found no substantive differences in findings across the two approaches. Therefore, we continue with the linear models, which give a fuller and more easily interpreted set of results. Second, we use survey weights in all analyses.

## Descriptive Results

**Figure 1:**
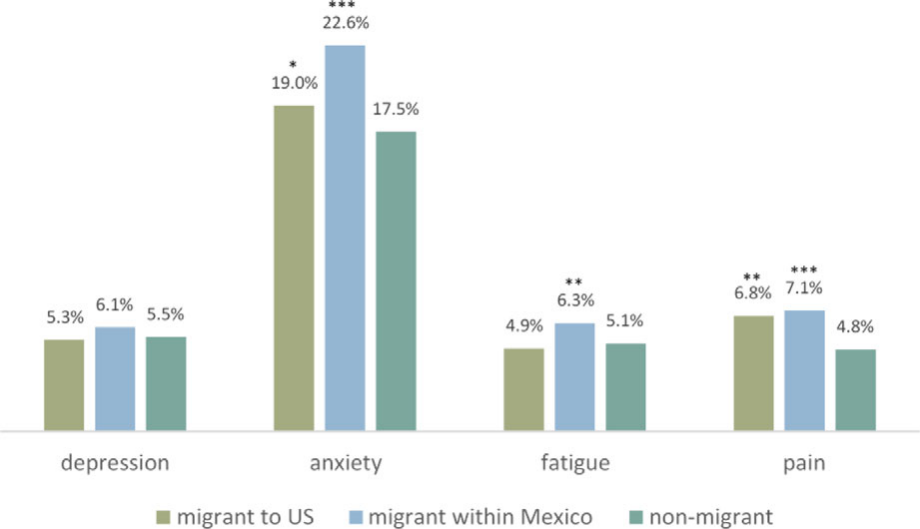
Mental health conditions of U.S. and Mexican Migrants and Non-Migrants. Significance tests of differences between migrants and non-migrants, * p < 0.10, ** p < 0.05, *** p < 0.01. Source: EDER 2017; ENH 2017.

We begin by describing differences in the mental health of U.S. migrants, internal migrants, and non-migrants. Along these lines, **Figure 1** presents two key findings. First, there are significant differences across groups in three of the four outcomes: self-reported anxiety, fatigue and pain. Compared to 17.5% of non-migrants who reported being anxious, substantially higher shares of U.S. migrants and Mexican internal migrants reported anxiety (19.0% and 22.6%, respectively). Compared to non-migrants, internal migrants reported more fatigue and both U.S. migrants and internal migrants reported more pain. Second, there are no differences in depression by migrant status.

**Table 1** presents descriptive statistics for the variables likely related to the mental health outcomes we observe by migrant status. Those who migrated to the United States tend to be older at the time of the survey than non-migrants and domestic migrants (39.1 vs. 35.3 and 37.1 years, respectively). Non-migrants are more likely to be female than either U.S. or internal migrants. This is especially the case for U.S. migrants, a full three quarters of whom are men. Yet, U.S. migrants distinguish themselves among the other groups in their propensity to be less educated. Among U.S. migrants, approximately 13.5% reported having a bachelor's degree compared to 21.9% of non-migrants and 22.3% of domestic migrants. U.S. migrants are less likely to be indigenous, but more likely to be employed and currently married. A smaller share of U.S. migrants reported living with a family member who was not a parent, spouse, or child, compared to domestic migrants and non-migrants. However, all three groups reported similar numbers of children living in the home. Migrants report less wealth than those who have never migrated, especially U.S. migrants who report less wealth than internal migrants and non-migrants. Among U.S. and domestic migrants, more than three-quarters report some migration experience longer than one year. Among U.S. migrants, 83.8% also migrated to a Mexican destination at some point in their lives. Among domestic migrants, 14.9% also migrated to a destination in the United States.

**Table 1:** Descriptive statistics by migration status and destination (unweighted).

	Non-migrant	Mexican Migrant	US Migrant	Total
Age (years)	35.3	37.1	39.1	36.0
Female (%)	58.0	51.1	29.0	54.9
Education				
No schooling (%)	3.0	2.2	1.4	2.6
Primary school (%)	18.7	18.3	24.6	18.5
Junior high (%)	31.7	31.9	37.8	31.9
High school (%)	23.3	22.8	21.2	23.0
College (%)	21.9	22.3	13.5	22.1
Graduate school (%)	1.4	2.6	1.5	1.9
Indigenous (%)	10.8	11.5	8.2	11.0
Employed (%)	71.3	77.2	82.7	73.7
Marital status				
Single, never married (%)	24.5	17.3	12.1	21.6
Currently married (%)	61.1	64.3	68.2	62.4
Formerly married (%)	14.4	18.5	19.6	16.0
Lives with other relative (%)	4.3	3.1	2.6	3.8
Number of children in house	1.3	1.3	1.4	1.3
Wealth index	0.044	-0.052	-0.212	0.000
Short migration (%)	NA	26.8	30.1	11.4
Long migration (%)	NA	85.2	88.0	34.2
Migrated to both US and MX (%)	NA	14.9	83.8	5.9
N	14,088	9,441	1,680	23,831
N (weighted)	36,900,000	21,300,000	3,465,266	58,900,000

NA, Not applicable.

Source: EDER 2017.

Given the differences between migrants and non-migrants, as well as between domestic and U.S. migrants, we explore below whether and how the differences are explained by age, gender, education, and other characteristics. To understand differences in the demographic profiles of each migrant group, below we explore the extent to which differences in **Figure 1** are explained by the variables presented in **Table 1**.

## Multivariate Results

The regression models analyze how the likelihood of depression, anxiety, chronic fatigue, and pain is associated with respondents' personal characteristics. Positive coefficients mean that higher values of explanatory variables are associated with greater likelihood of experiencing one of the four mental health conditions; negative coefficients mean that higher values of explanatory variables are associated with lower likelihoods of experiencing these conditions.

We begin with coefficients in the bottom rows of **Table 2** derived from the OLS models predicting self-reported depression, anxiety, fatigue, and pain. The migration coefficients in **Table 2** reveal no significant differences between U.S. return migrants and non-migrants in the four outcomes. However, coefficients for domestic migration in Mexico are positive and significant for three of the four self-reported mental health symptoms. Having migrated within Mexico is positively related to anxiety, fatigue, and pain, meaning domestic migrants report higher levels of these symptoms net of other model characteristics. This pattern is consistent with that observed in **Figure 1**, suggesting that the higher rates of anxiety, fatigue, and pain among domestic migrants relative to non-migrants are not explained by differences in the two group's characteristics. Domestic migrants have 4.5% greater probability of anxiety, 1.3% greater probability of fatigue, and 2.0% greater probability of pain than non-migrants, net of other attributes. Thus, migration within Mexico appears associated with poorer mental health, relative to non-migrants.

**Table 2:** OLS models predicting self-reported depression, anxiety, fatigue, and pain.

	(1)	(2)	(3)	(4)
	Depression	Anxiety	Fatigue	Pain
Age	0.000	0.005	-0.006***	-0.007***
	(0.002)	(0.003)	(0.002)	(0.002)
Age squared	0.000	-0.000	0.000***	0.000***
	(0.000)	(0.000)	(0.000)	(0.000)
Female	0.018***	0.034***	0.015***	0.011**
	(0.005)	(0.009)	(0.005)	(0.005)
Education (ref=no schooling)				
Primary school	-0.027	-0.000	-0.021	-0.013
	(0.020)	(0.025)	(0.018)	(0.019)
Junior high	-0.044**	0.002	-0.033*	-0.018
	(0.019)	(0.025)	(0.018)	(0.018)
High school	-0.062***	-0.008	-0.043**	-0.030
	(0.019)	(0.026)	(0.018)	(0.019)
College	-0.074***	-0.000	-0.037**	-0.034*
	(0.019)	(0.026)	(0.018)	(0.019)
Graduate school	-0.094***	0.030	-0.038	-0.050**
	(0.020)	(0.047)	(0.025)	(0.023)
Indigenous	-0.015**	-0.062***	-0.016**	0.007
	(0.007)	(0.012)	(0.007)	(0.009)
Employed	-0.024***	-0.001	0.006	-0.001
	(0.007)	(0.010)	(0.006)	(0.006)
Marital status (ref=single)				
Currently married	-0.011	0.032***	0.003	0.006
	(0.007)	(0.012)	(0.007)	(0.006)
Formerly married	0.039***	0.075***	0.012	0.028***
	(0.009)	(0.014)	(0.009)	(0.009)
Lives with other relative	-0.010	-0.036**	-0.003	-0.011
	(0.008)	(0.015)	(0.011)	(0.007)
Number of children in house	-0.003*	0.012***	-0.000	-0.001
	(0.002)	(0.004)	(0.002)	(0.003)
Wealth index	0.007***	0.007**	0.003	0.004**
	(0.002)	(0.003)	(0.002)	(0.002)
Migration (ref=nonmigrant)				
Migrated to US	-0.002	0.003	-0.009	0.010
	(0.016)	(0.029)	(0.017)	(0.020)
Migrated within Mexico	0.006	0.045***	0.013**	0.020***
	(0.005)	(0.009)	(0.005)	(0.005)
Migrated to both	-0.001	-0.041	-0.018	-0.024
	(0.018)	(0.033)	(0.019)	(0.021)
Constant	0.070*	-0.051	0.117***	0.119***
	(0.038)	(0.062)	(0.042)	(0.042)
Observations	23,731	23,719	23,795	23,787

*p < 0.10 **p < 0.05 ***p < 0.01 Heteroskedasticity-robust standard errors in parentheses. All models are estimated using a linear probability model and probability weights. Source: EDER 2017; ENH 2017.

Model 1 also reveals that gender, education, marital status, having children at home, and wealth are associated with self-reported depression controlling for other observable characteristics that relate to mental health. Women report more depression, and compared to those without education, having a junior high school or higher education is associated with less depression. Being indigenous and employed are also associated with less depression, but being formerly married, relative to being single, is related to higher reports of depression. Having more sons and daughters in the household is associated with slightly lower risks of depression, but having more wealth as measured in assets has a small positive association with depression.

Although many demographic and socioeconomic attributes associated with depression are also associated with anxiety, fatigue and pain, not all are. For example, age appears negatively associated with fatigue and pain, and the positive coefficients for age squared suggest that these negative age effects become less negative as people age. Education also matters, but differently for different mental health symptoms. Generally, higher levels of education are associated with lower likelihoods of depression, fatigue, and pain. Being indigenous is also associated with less depression, anxiety, and fatigue, although it is not significantly related to pain. Being employed is significantly and negatively related only to symptoms of depression.

Marital status is also associated with mental health symptoms. Compared to single respondents, those formerly married report significantly more depression, anxiety, and pain. Moreover, being currently married is also positively associated with anxiety. With respect to other relatives or children living in the household, we see that the former is negatively related, and the latter is positively associated, with anxiety. In addition, wealth as measured in household assets is significantly associated with depression, anxiety, and pain. Greater wealth translates into more of these symptoms.

Although the above models consider whether mental health outcomes are associated with migration and other characteristics, they assume that the relationships between factors—such as age or gender—and mental health are similar for migrants and non-migrants. For example, migrant vs. non-migrant differences in mental health may be related to the fact that migrants are older than non-migrants (see **Table 1**) or to migrant vs. non-migrant *differences in the effects of age on mental health*. To examine this possibility, we decompose migrant-non-migrant differences in the four outcomes into three parts: (1) those explained by differences in characteristics (often referred to as differences in endowments); (2) those explained by differences in the returns, e.g., coefficients, to these characteristics; and (3) those explained by interactions between characteristics (such as age) and their effects (41–43).^7^ If we find that migrant vs. non-migrant differences in mental health are mostly due to differences in characteristics, this suggests migrant selectivity. Alternatively, if we find that migrant vs. non-migrant differences in mental health are mostly due to differences in the coefficients, this suggests that, even if the groups had the same characteristics, we would still see mental health differences that may result from migration.

**Table 3** presents the decomposition results. For each dependent variable, there are two columns: the first refers to migrants within Mexico and the second refers to U.S. migrants. For each, we present the average prevalence of each mental health condition for migrants and non-migrants, and then the difference between the two groups. We begin with the depression model, and see that the findings are consistent with those found in **Table 2**. For those who migrated within Mexico (Column 1a), the third row shows that the difference in the prevalence of depression between migrants and non-migrants is -.006 and not significant. In the depression model for those with U.S. migrant experience (Column 1b), the difference in depression is also small and not significant (-.003).

**Table 3:** Oaxaca-Blinder decompositions.

	(1a)	(1b)	(2a)	(2b)	(3a)	(3b)	(4a)	(4b)
	Depression		Anxiety		Fatigue		Pain	
	Mexico	US	Mexico	US	Mexico	US	Mexico	US
Prevalence for non-migrants	0.055***	0.055***	0.175***	0.175***	0.051***	0.051***	0.048***	0.048***
	(0.003)	(0.003)	(0.005)	(0.005)	(0.003)	(0.003)	(0.003)	(0.003)
Prevalence for migrants	0.061***	0.058***	0.226***	0.198***	0.063***	0.043***	0.071***	0.064***
	(0.004)	(0.007)	(0.007)	(0.012)	(0.004)	(0.006)	(0.004)	(0.007)
Difference (migrants-nonmigrants)	-0.006	-0.003	-0.050***	-0.022*	-0.012**	0.008	-0.023***	-0.016**
	(0.005)	(0.007)	(0.008)	(0.013)	(0.005)	(0.006)	(0.005)	(0.008)
Due to endowments	0.003	0.012	-0.005	0.021	-0.000	0.001	-0.004*	0.003
	(0.002)	(0.015)	(0.003)	(0.028)	(0.002)	(0.014)	(0.002)	(0.017)
Due to coefficients	-0.004	-0.000	-0.040***	-0.005	-0.009*	0.013*	-0.018***	-0.008
	(0.005)	(0.007)	(0.008)	(0.014)	(0.005)	(0.007)	(0.005)	(0.008)
Due to interaction	-0.004**	-0.015	-0.006	-0.038	-0.003	-0.005	-0.001	-0.011
	(0.002)	(0.015)	(0.004)	(0.029)	(0.002)	(0.014)	(0.002)	(0.017)
% due to endowments	-50.0	-400.0	10.0	-95.5	0.0	12.5	17.4	-18.8
% due to coefficients	66.7	0.0	80.0	22.7	75.0	162.5	78.3	50.0
% due to interaction	66.7	500.0	12.0	172.7	25.0	-62.5	4.3	68.8
								
N	23,429	15,699	23,418	15,693	23,493	15,746	23,485	15,737

*p < 0.10 **p < 0.05 ***p < 0.01 Heteroskedasticity-robust standard errors in parentheses. All models are estimated separately using a Oaxaca-Blinder decomposition and probability weights and control for gender, age, age squared, education, indigenous, employed, marital status, living with other relatives, number of children in the house, and the wealth index. See Appendix for full technical details. Percentages may not always sum to 100% due to rounding error.

Source: EDER 2017; ENH 2017.

However, in the remaining columns presented in **Table 3**, we see significant non-migrant vs. migrant differences in anxiety, fatigue and pain.^8^ With respect to differences in anxiety between those who have migrated within Mexico and non-migrants (Column 2a), the difference is -.050, indicating that the prevalence of anxiety among internal migrants in Mexico is 5.0 percentage points greater than it is for non-migrants. This difference is decomposed into three sets of differences due to (1) the socioeconomic and demographic characteristics of the groups, which age, gender, and the other variables in **Table 2** (usually referred to as endowments); (2) the relationships – as captured in coefficients -- between a group's characteristics and the mental health symptoms we measure (usually referred to as returns to characteristics); and (3) the interactions between characteristics and returns to these characteristics.

In this example, we see that -0.005 of the -.050 difference is explained by endowments, -.040 is explained by coefficients and -.006 is explained by the interaction between endowments and coefficients. Interpreting these as percentages, the difference due to endowments amounts to 10%, while returns to those endowments explain 80% of the difference. The difference due to interaction between endowments and coefficients is 12%.

By comparison, the difference in anxiety between those with U.S. experience and non-migrants is also significant (-.022), but the decomposition suggests that the difference due to their demographic profiles (characteristics) acts in the opposite direction of the observed difference, almost entirely offsetting it. This means that, based on their characteristics alone, we would expect migrants with U.S. experience to have almost no difference from non-migrants. The higher prevalence of anxiety among U.S. migrants derives entirely from the interaction between their characteristics and the relationship of these characteristics to mental health or, in other words, the unique relationship between migrants' demographic profiles and their mental health.

The decompositions for fatigue and pain reveal fairly consistent results among internal migrants. Among internal migrants, prevalence of fatigue and pain that are 1.2 and 2.3 percentage points higher than non-migrants. Decomposition results suggest that substantially more of these differences is explained by returns to the characteristics controlled for in the models rather than due to endowments (or selectivity). Similarly, decomposition of the U.S. migrants and non-migrant difference in pain also reveals that it derives more from differences in the relationship of the characteristics to mental health in the model than from the prevalence of these characteristics in the groups, that is, from selectivity.

Finally, as suggested above, it is important to note that in the models for anxiety, fatigue, and pain, when differences between migrants and non-migrants are significant, these differences cannot be fully explained by differences in migrant and non-migrant characteristics. In one case, the percent of the difference explained by endowments is negative, indicating that the characteristics of U.S. migrants align with their being better off (with lower anxiety) than non-migrants, and that migrants are self-selected on characteristics associated with lower anxiety. In other cases, the share of the difference between migrants and non-migrants is less than 100%, meaning that not all of the migrant vs. non-migrant difference is explained by model characteristics. This suggests that differences in rates of some mental health conditions would be substantially lower, if not reversed in direction, if migrants experienced the same coefficients on their characteristics as non-migrants.

## Discussion and Conclusion

In 2017, Mexican young adults—between the ages of 20 and 55—reported relatively high levels of depression, anxiety, fatigue, and pain. One in 20 Mexican young adults reported depression, fatigue, and pain, and one in five reported anxiety. This prevalence of mental health problems varies among Mexican young adults by a number of characteristics, including gender, age, and education. We found that mental health problems also vary by migrant status in Mexico. Both returned U.S. migrants and internal migrants had higher rates of anxiety and pain than non-migrants, and internal migrants also had higher rates of fatigue.

Internal migrants' higher rates of anxiety, fatigue, and pain, compared to non-migrants, hold even after we control for observed differences between groups, such as that internal migrants are older, more likely to be men, and slightly better educated than non-migrants. The domestic-migrant-non-migrant difference appears to arise from how these characteristics are associated with anxiety, fatigue, and pain, rather than from differences between the groups in those characteristics. This implies that, if migrants experienced the same mental health implications of their demographic characteristics as non-migrants, we would see substantially lower rates of depression, anxiety, fatigue, and pain for domestic migrants and nearly no difference between domestic migrants and non-migrants.

The story for U.S. migrants is more complex. Although the descriptive findings in **Figure 1** show that U.S. migrants have higher rates of anxiety and pain than non-migrants, **Table 2** reveals these differences do not remain after controlling for relevant characteristics. Thus, when differences in observed socio-economic and demographic characteristics such as age and gender are controlled, U.S. migrants self-reported symptoms related to depression, anxiety, fatigue, and pain are comparable to non-migrants. However, once we allow the relationship between each observed characteristic and the mental health outcome to vary for migrants and non-migrants, we again observe a disadvantage for U.S. migrants in terms of anxiety and pain. This arises largely because the returns to characteristics—or the ways that characteristics associate with self-reported mental health symptoms—are more deleterious for migrants than for non-migrants. In other words, U.S. migrants are selected on characteristics associated with good mental health—they are positively selected—but those characteristics are not protective for them. This could suggest a deleterious effect of migration that is unexplained by the variables included in the models.

As with all studies, we caution readers to consider several limitations of this analysis. First, one weakness of the data set we use is that it contains data on returned U.S. migrants, but for those migrating in Mexico, the data identify those migrants who have not returned. Migrants who return may differ from those who have not, and these differences may be associated with mental health. For example, migrants who develop a mental health condition or who are exposed to psychological trauma associated with a mental health disorder may be more likely to return to their origins. Arenas et al. (44), for example, found some evidence for this point: poor physical and mental health were associated with a U.S. migrant's probability of return to Mexico but the mental health – return relationship was not statistically insignificant. If migrants who develop mental health disorders are more likely to return, then our analysis will overestimate the relationship between migration and mental health. On the other hand, migrants who return may feel social pressure to report a positive migration experience. If this occurred, then the relationship between migration and mental health would be understated.

A second limitation derives from our use of self-reported mental health conditions. As mentioned earlier, these self-reports may or may not directly correspond to underlying mental health conditions and disorders. In addition, because stigma is often linked to mental health underreporting, this analysis is likely to underestimate the relationship between mental health and migration. A third limitation concerns endogeneity; that is, migration may be an explanatory variable of mental health but it may also be the consequence. Thus, migration is not a random exogenous determinant of mental health but may itself be driven by the same shock that is related to mental health. Relatedly, this analysis does not consider the amount of time that has passed between the migration event and the time of the survey, during which other endogenous shocks may have occurred or the casual mechanism between migration and mental health may have weakened. In the sample considered, the average years since last migration is 5.8 and the maximum is 50. Although the results presented above are, in general, robust to limiting the sample to those who have migrated within the last five years (analysis available upon request), this reduces significance because it reduces the sample size.

Our analysis suggests that future researchers must consider fully interacted models as they consider migrant differences in mental health. For example, if we only estimated the regression models (seen in **Table 2**), then we would conclude that differences between U.S. migrants and non-migrants are fully explained by their characteristics. However, once we consider how their characteristics relate differently to mental health, as seen in the decomposition results (**Table 3**), the difference between U.S. migrants and non-migration re-emerges.

Migration is a costly and risky endeavor that tends to select on people with skills, capacities, and resources to invest in a better future. Understanding the balance of these forces—of selection of hardier, more ambitious, and potentially more mentally healthy people—into a migration flow, combined with its costs and benefits for mental health, is a necessary challenge for future research. Assuming Mexico-U.S. migration continues to abate and Mexico's trajectory is as both a host and transit country for Central Americans and others seeking protection and economic security, then mental health issues are likely to intensify especially if domestic and transit migrants encounter violent conditions as they travel northward.

## Data Availability Statement

The datasets generated for this study are available on request to the corresponding author.

## Ethics Statement

In this study, ethical approval and written informed consent were not required because the study used de-identified publicly available data from the following website: https://www.inegi.org.mx/programas/eder/2017/.

## Author Contributions

In this collaboration, KD led the investigation by supervising the data analysis and writing the first and final drafts of the manuscript. EH consulted on the analysis and the draft, contributing to both. LC generated the analysis and also contributed to writing the first and final drafts.

## Conflict of Interest

The authors declare that the research was conducted in the absence of any commercial or financial relationships that could be construed as a potential conflict of interest.

The reviewer KH declared a shared affiliation, with no collaboration, with several of the authors KD and LC to the handling Editor.

## Footnotes

1 Because of space limitations, we only review studies that focus on Mexican migration and mental health. To understand the mental health of refugees and other forced migrants, see Belz et al. (13); Chen et al. (14); Groen et al. (15); Opaas and Varvin (16); and Verroken et al. (17).

2 Many studies use small select U.S. samples, such as Arbona et al. (27), who report that fear of deportation was associated with extra- and intra-familial acculturative stress among Mexican and Central American immigrants in two Texas cities.

3 If we relied only on the EDER, we would have had to use questions about mental health disability; these are limited to very serious events that disabled persons report having for at least a year.

4 We excluded from our analytic sample 30 respondents who reported migrating to an international destination that was not the United States.

5 For more details about variable construction, see **Table A1**.

6 Although correlated with mental health, fatigue and pain may also relate to a variety of socio-biological causes such as overworking, insomnia, malnutrition or other unattended medical conditions.

7 Note that **Tables A2**–**A5** present the weighted means and multivariate models upon which the decompositions are based.

8 There is one exception. In the fatigue model for U.S. migrants, the difference between migrants and non-migrants is small and not significant (.008).
