# Supplementary material for: Migration and Mental Health in Mexico: Domestic Migrants, Return U.S. Migrants, and Non-Migrants
Source: Front Psychiatry. 2020 Feb 3;10:970. doi: 10.3389/fpsyt.2019.00970 (PMC7008711; doi:10.3389/fpsyt.2019.00970)
Supplement: Supplementary file 1 [file DataSheet_1.docx]

**Methodological Appendix: Oaxaca-Blinder Decompositions**

This appendix describes the methods for calculating the Oaxaca-Blinder decompositions presented in the main text (Tables 3-6) (Oaxaca 1973; Blinder 1973; Jann 2008). This method has been used extensively for estimating wage gaps, such as the gender wage gap. We generated the analysis in this paper using the Stata command **oaxaca** (Jann 2008).

Simplifying from the regression models above, the mental health of each individual *i* belonging to group *j* (migrant or non-migrant) is as follows:

$$mentalhealth_{\mathrm{ij}}=\boldsymbol{\delta}_{\boldsymbol{j}}\boldsymbol{X}_{\boldsymbol{ij}}+\omega_{ij}$$

where ***X_ij_­*** represents a vector of individual characteristics.

Taking the difference of these equations for the groups of non-migrants and migrants:

$$mentalhealth_{nonmig}- mentalhealth_{mig}= \delta_{nonmig}\bar{X}_{nonmig}- \delta_{mig}\bar{X}_{mig}$$

which can be rearranged:

$$mentalhealth_{nonmig}- mentalhealth_{mig}= \delta_{mig}\left( \bar{X}_{nonmig}-\bar{X}_{mig} \right)+\left( \delta_{nonmig}- \delta_{mig} \right)\bar{X}_{mig}$$

$$+(\delta_{nonmig}-\delta_{mig})(\bar{X}_{nonmig}-\bar{X}_{mig})$$

where the first term of the right-hand side represents the portion explained by differences in endowments, the second represents the differences in coefficients, and the third represents the difference due to interactions between endowments and coefficients. For multivariate models, the total difference due to each is the sum of the differences of the respective terms for each covariate. For a model with a constant, the difference in constants is added to the unexplained portion, since this is a difference that is not related to differences in the endowments.

The first step to calculating the Oaxaca-Blinder decomposition is to estimate regressions with the full set of covariates, splitting the sample across the groups to be compared. In this case, we split the sample by type of migration, so that we can compare U.S. migrants and migrants within Mexico with non-migrants. Table A3 presents the full results of these regressions.

Next, we calculated the differences due to endowments, coefficients, and interactions using the formula above, for each covariate. For example, to calculate the difference in depression between U.S. migrants and non-migrants associated with age, we calculate (drawing the means of age from the weighted statistics reported in Table A2)^[[1]](#footnote-1)^:

Difference due to endowments = -0.0025 * (34.8-39.1) = 0. 011 Difference due to coefficients = (-0.0010 + 0.0025) * 39.1 = 0.059

Difference due to interaction = (-0.0010 + 0.0025) * (34.8-39.1) = -0.007

Similarly, for depression for migrants to Mexico, the difference associated with employment:

Difference due to endowments = -0.0385* (0.703-0.757) = 0.0021

Difference due to coefficients = (-0.0161 + 0.0385) * 0.757 = 0.0170

Difference due to interaction = (-0.0161 + 0.0385) * (0.703-0.757) = -0.00121

These differences due to endowments, coefficients, and their interaction replicate (with rounding error) the findings from the Oaxaca-Blinder decompositions shown in Table A4 and Table A5.

The final step in computing the Oaxaca-Blinder decomposition is to sum (separately) differences due to endowments, coefficients, and interactions across covariates. This gives the total difference due to each. For depression for U.S. migrants, the total difference:

Due to endowments is 0.0130;

Due to coefficients is -0.002; and

Due to interaction is -0.018.

These replicate (with rounding error) the results from the main text (Table 3).

| **Table A1: Variable Definitions** | | |
| --- | --- | --- |
| Variable name | English definition | EDER question |
| Age | The respondent’s age (in years) at the time of survey. | *¿En qué mes y año nació usted?* |
| Female | A binary variable for whether the respondent is male or female. | *(NOMBRE) es … Hombre o Mujer* |
| No education | A binary variable for whether the respondent has no education (used as the reference group in regressions). | *¿Cuál es el último año o grado que aprobó (NOMBRE) en la escuela?* |
| Primary or less | A binary variable for whether the respondent has at most primary education (*Preescolar* or *Primaria*). |  |
| Junior high or less | A binary variable for whether the respondent has at most junior high education (*secundaria*, *normal bàsica*, or *Estudios técnicos o comerciales con primaria terminada*). |  |
| High school or less | A binary variable for whether the respondent has at most completed high school education (*Preparatoria o bachillerato* or *Estudios técnicos o comerciales con secundaria terminada*). |  |
| Bachelor's degree / licentiate | A binary variable for whether the respondent has at most completed a Bachelor’s degree or license (*Normal de licenciatura, Estudios técnicos o comerciales con preparatoria terminada,* or *Licenciatura o profesional*). |  |
| Post-graduate | A binary variable for whether the respondent has at most completed a postgraduate degree (*Maestría* or *Doctorado*). |  |
| Indigenous | A binary variable for whether the respondent belongs to an indigenous people. | *¿Pertenece a un pueblo indígena?* |
| Employed | A binary variable for whether the respondent has worked in the last week. | *Durante la semana pasada, ¿(NOMBRE) trabajó?* |
| Never married | A binary variable for whether the respondent has never been married (used as reference group in regressions). | *¿A qué edad o en qué año se unió con (NOMBRE)?*  *Y cuando comenzó esta unión, ¿era… ¿En algún momento de su unión con (NOMBRE) se*  *casaron por lo civil, religioso o ambos? ¿Usted sigue unido(a) o casado(a) con (NOMBRE)?* |
| Currently married | A binary variable for whether the respondent is currently married or in a civil or religious partnership. |  |
| Formerly married | A binary variable for whether the respondent has been divorced, separated, or widowed. |  |
| Lives with other relative | A binary variable for whether the respondent has lived with another family member (not including immediate family) in the last three years. | *Dígame los periodos de al menos un año durante los cuales usted y alguna otra persona de su familia de origen (abuela, abuelo, primo, tío, etcétera) vivieron juntos.* |
| Number of children in household | The number of sons or daughters of the respondent in the household. | *¿Podría decirme todos los periodos, de al menos un año, durante los cuales usted y (NOMBRE) vivieron juntos?* |
| Wealth index | An index generated from a principal components analysis (PCA) of asset ownership for the following assets: video player, DVD, washing machine, and microwave. | *Podría decirme si en esta vivienda cuentan con… videocasetera, reproductor de DVD, lavadora en el hogar, microondas.* |
| Short migration | A binary variable for whether the individual reported having migrated for less than one year. | *Alguna vez en su vida, ¿cambió de domicilio a otras localidades por un tiempo menor a un año?* |
| Long migration | A binary variable for whether the individual lived in another country or state within Mexico for more than one year. This is defined by comparing to the person’s birthplace. | *Contiene la ubicación geográfica retrospectiva*  *de las personas. Los tres primeros dígitos representan la clave del país, los siguientes*  *dos dígitos representan la clave de la entidad y los siguientes tres la clave del municipio. Éstas corresponden al Catálogo de claves utilizado*  *por el INEGI.* |
| Ever migrated | A binary variable for whether the individual has either short migration or long migration. |  |
| Ever migrated to US | A binary variable for whether the individual has ever migrated (short or long) to the United States. |  |
| Ever migrated to Mexico | A binary variable for whether the individual has ever migrated (short or long) to Mexico. |  |
| Depression | A binary variable for whether the individual feels depressed on a weekly or daily basis. | *¿Con qué frecuencia (NOMBRE) se siente deprimido?* |
| Anxiety | A binary variable for whether the individual feels nervous on a weekly or daily basis. | *¿Con qué frecuencia (NOMBRE) se ha sentido preocupado o nervioso?* |
| Fatigue | A binary variable for whether the individual feels fatigued most days or every day. | *¿En los últimos 3 meses, ¿con qué frecuencia (NOMBRE) se ha sentido muy cansado o exhausto?* |
| Pain | A binary variable for whether the individual feels pain most days or every day. | *¿En los últimos 3 meses, con qué frecuencia (NOMBRE) ha sentido dolor?* |

| Table A2: Descriptive statistics, weighted | | | | |
| --- | --- | --- | --- | --- |
|  | Non-Migrant | Mexican Migrant | US Migrant | Total |
| Age | 34.8 | 37.0 | 39.1 | 35.6 |
| Age squared | 1310.2 | 1465.4 | 1601.8 | 1369.5 |
| Female (%) | 57.7 | 51.8 | 28.0 | 55.2 |
| Education |  |  |  |  |
| No schooling (%) | 2.6 | 2.2 | 1.8 | 2.4 |
| Primary school or less (%) | 18.5 | 17.8 | 25.9 | 18.3 |
| Junior high or less (%) | 30.3 | 30.4 | 36.2 | 30.4 |
| High school or less (%) | 23.8 | 24.1 | 20.0 | 23.9 |
| Bachelor's degree (%) | 23.4 | 22.7 | 13.9 | 23.1 |
| Masters or more (%) | 1.3 | 2.8 | 2.2 | 1.9 |
| Indigenous (%) | 10.0 | 11.3 | 7.7 | 10.4 |
| Employed (%) | 70.3 | 75.7 | 84.6 | 72.4 |
| Marital status | |  |  |  |
| Single, never married (%) | 26.5 | 16.5 | 10.8 | 22.8 |
| Currently married (%) | 62.8 | 69.6 | 73.7 | 65.3 |
| Formerly married (%) | 10.7 | 13.8 | 15.5 | 11.9 |
| Lives with other relative (%) | 4.5 | 3.3 | 2.2 | 4.0 |
| Number of children in house | 1.3 | 1.37 | 1.49 | 1.3 |
| Wealth index | -0.062 | -0.191 | -0.273 | -0.113 |
| Short migration (%) | NA | 24.5 | 28.9 | 9.6 |
| Long migration (%) | NA | 86.2 | 88.2 | 31.7 |
| Migrated to both (%) | NA | 13.5 | 82.9 | 4.9 |
| N | 14,088 | 9,441 | 1,680 | 23831 |
| N (weighted) | 3.69E+07 | 2.13E+07 | 3,465,266 | 58,900,000 |

| Table A3: Fully interacted models | | | | | | |
| --- | --- | --- | --- | --- | --- | --- |
|  | (1a) | (1b) | (1c) | (2a) | (2b) | (2c) |
|  | Depression | | | Anxiety | | |
|  | non-migrant | US | Mexico | non-migrant | US | Mexico |
| Age | -0.001 | -0.003 | 0.002 | 0.002 | -0.003 | 0.010 |
|  | (0.003) | (0.006) | (0.003) | (0.004) | (0.012) | (0.006) |
| Age squared | 0.000 | 0.000 | -0.000 | 0.000 | 0.000 | -0.000 |
|  | (0.000) | (0.000) | (0.000) | (0.000) | (0.000) | (0.000) |
| Female (ref=Male) | 0.019*** | 0.055*** | 0.015* | 0.037*** | 0.075** | 0.026* |
|  | (0.006) | (0.021) | (0.009) | (0.010) | (0.032) | (0.015) |
| Education (ref=no schooling) | | |  |  |  |  |
| Primary school or less | -0.038 | 0.016 | -0.012 | 0.010 | 0.034 | -0.027 |
|  | (0.024) | (0.041) | (0.037) | (0.029) | (0.090) | (0.050) |
| Junior high or less | -0.056** | 0.006 | -0.031 | 0.003 | -0.002 | -0.008 |
|  | (0.023) | (0.040) | (0.035) | (0.029) | (0.089) | (0.049) |
| High school or less | -0.067*** | -0.013 | -0.058 | -0.005 | 0.038 | -0.025 |
|  | (0.023) | (0.042) | (0.035) | (0.029) | (0.091) | (0.050) |
| Bachelor's degree | -0.077*** | -0.039 | -0.072** | -0.010 | 0.084 | 0.004 |
|  | (0.023) | (0.043) | (0.035) | (0.030) | (0.098) | (0.051) |
| Masters or more | -0.108*** | -0.057 | -0.076** | 0.024 | -0.118 | 0.027 |
|  | (0.024) | (0.051) | (0.035) | (0.072) | (0.097) | (0.071) |
| Indigenous (ref=non-indigenous) | -0.022*** | 0.015 | -0.004 | -0.059*** | -0.013 | -0.063*** |
|  | (0.008) | (0.024) | (0.012) | (0.015) | (0.041) | (0.018) |
| Employed (ref=not employed) | -0.016** | -0.001 | -0.038*** | 0.014 | -0.034 | -0.030 |
|  | (0.007) | (0.020) | (0.013) | (0.012) | (0.036) | (0.018) |
| Marital status (ref=single) | | |  |  |  |  |
| Currently married | 0.002 | -0.040* | -0.035** | 0.035** | 0.014 | 0.025 |
|  | (0.007) | (0.022) | (0.017) | (0.014) | (0.046) | (0.023) |
| Formerly married | 0.038*** | 0.047 | 0.037** | 0.070*** | 0.034 | 0.084*** |
|  | (0.011) | (0.033) | (0.019) | (0.018) | (0.050) | (0.026) |
| Lives with other relative (ref=not) | -0.009 | -0.053** | -0.012 | -0.031* | -0.140*** | -0.051** |
|  | (0.009) | (0.023) | (0.016) | (0.018) | (0.042) | (0.026) |
| Number of children in house | -0.006*** | 0.005 | 0.002 | 0.010** | 0.015 | 0.014** |
|  | (0.002) | (0.005) | (0.003) | (0.004) | (0.011) | (0.006) |
| Wealth index | 0.006*** | 0.008 | 0.009*** | 0.005 | 0.024** | 0.009* |
|  | (0.002) | (0.006) | (0.003) | (0.004) | (0.010) | (0.005) |
| Migrated to both | -- | 0.001 | -0.004 | -- | 0.004 | -0.035** |
|  |  | (0.016) | (0.009) |  | (0.032) | (0.017) |
| Constant | 0.083* | 0.043 | 0.062 | -0.013 | 0.083 | -0.045 |
|  | (0.048) | (0.112) | (0.064) | (0.072) | (0.241) | (0.119) |
| Observations | 14,026 | 1,673 | 9,403 | 14,023 | 1,670 | 9,395 |
| R-squared | 0.025 | 0.038 | 0.037 | 0.027 | 0.030 | 0.022 |
| * p<0.10 ** p<0.05 *** p<0.01 Heteroskedasticity-robust standard errors in parentheses. All models are estimated using a linear probability model and probability weights.  Source: EDER 2017; ENH 2017 | | | | | | |

| Table A3: Fully interacted models (continued) | | | | | | |
| --- | --- | --- | --- | --- | --- | --- |
|  | (3a) | (3b) | (3c) | (4a) | (4b) | (4c) |
|  | Fatigue | | | Pain | | |
|  | non-migrant | US | Mexico | non-migrant | US | Mexico |
| Age | -0.006* | 0.001 | -0.008** | -0.007*** | -0.011* | -0.008* |
|  | (0.003) | (0.005) | (0.004) | (0.002) | (0.007) | (0.004) |
| Age squared | 0.000*** | 0.000 | 0.000** | 0.000*** | 0.000* | 0.000*** |
|  | (0.000) | (0.000) | (0.000) | (0.000) | (0.000) | (0.000) |
| Female | 0.017*** | 0.013 | 0.013 | 0.009 | 0.027 | 0.016* |
|  | (0.006) | (0.013) | (0.009) | (0.006) | (0.019) | (0.009) |
| Education (ref=no schooling) |  |  |  |  |  |  |
| Primary school or less | -0.007 | -0.002 | -0.049 | -0.007 | -0.009 | -0.030 |
|  | (0.020) | (0.050) | (0.038) | (0.017) | (0.066) | (0.043) |
| Junior high or less | -0.027 | -0.014 | -0.049 | -0.009 | -0.039 | -0.037 |
|  | (0.019) | (0.048) | (0.037) | (0.017) | (0.065) | (0.042) |
| High school or less | -0.029 | -0.023 | -0.072* | -0.015 | -0.052 | -0.059 |
|  | (0.019) | (0.049) | (0.037) | (0.017) | (0.065) | (0.044) |
| Bachelor's degree | -0.026 | -0.025 | -0.060 | -0.020 | -0.076 | -0.064 |
|  | (0.019) | (0.049) | (0.038) | (0.018) | (0.065) | (0.044) |
| Masters or more | -0.016 | -0.045 | -0.068 | -0.021 | -0.104 | -0.088* |
|  | (0.035) | (0.052) | (0.042) | (0.031) | (0.068) | (0.045) |
| Indigenous | -0.021** | -0.017 | -0.009 | 0.011 | 0.014 | 0.000 |
|  | (0.008) | (0.018) | (0.013) | (0.012) | (0.026) | (0.014) |
| Employed | 0.004 | -0.010 | 0.011 | 0.001 | -0.023 | -0.004 |
|  | (0.007) | (0.015) | (0.010) | (0.006) | (0.023) | (0.012) |
| Marital status (ref=single) |  |  |  |  |  |  |
| Currently married | -0.003 | 0.005 | 0.017 | 0.004 | 0.021 | 0.013 |
|  | (0.008) | (0.014) | (0.012) | (0.008) | (0.019) | (0.011) |
| Formerly married | 0.009 | 0.017 | 0.022 | 0.027** | 0.015 | 0.034** |
|  | (0.012) | (0.018) | (0.014) | (0.012) | (0.023) | (0.015) |
| Lives with other relative | -0.000 | -0.029*** | -0.009 | -0.012** | -0.042*** | -0.007 |
|  | (0.014) | (0.009) | (0.017) | (0.006) | (0.013) | (0.018) |
| Number of children in house | 0.001 | -0.002 | -0.001 | 0.000 | -0.004 | -0.002 |
|  | (0.003) | (0.006) | (0.004) | (0.003) | (0.007) | (0.004) |
| Wealth index | 0.002 | 0.006 | 0.004 | 0.003* | 0.001 | 0.005* |
|  | (0.002) | (0.005) | (0.003) | (0.002) | (0.005) | (0.003) |
| Migrated to both | -- | -0.007 | -0.029*** | -- | -0.004 | -0.016 |
|  |  | (0.018) | (0.008) |  | (0.021) | (0.010) |
| Constant | 0.099** | -0.004 | 0.177** | 0.101** | 0.230* | 0.158* |
|  | (0.049) | (0.099) | (0.078) | (0.042) | (0.132) | (0.092) |
| Observations | 14,067 | 1,679 | 9,426 | 14,057 | 1,680 | 9,428 |
| R-squared | 0.018 | 0.015 | 0.019 | 0.025 | 0.026 | 0.037 |
| * p<0.10 ** p<0.05 *** p<0.01 Heteroskedasticity-robust standard errors in parentheses. All models are estimated using a linear probability model and probability weights.  Source: EDER 2017; ENH 2017 | | | | | | |

| Table A4: Full Oaxaca-Blinder Decomposition Results for US migrants | | | | | |  |  |  |  |  |  |  |
| --- | --- | --- | --- | --- | --- | --- | --- | --- | --- | --- | --- | --- |
|  | (1) | | | (2) | | | (3) | | | (4) | | |
|  | depression | | | anxiety | | | fatigue | | | pain | | |
| Difference due to: | endow | coef | inter | endow | coef | inter | endow | coef | inter | endow | coef | inter |
| Age | 0.011 | 0.061 | -0.007 | 0.013 | 0.176 | -0.020 | -0.004 | -0.260 | 0.029 | 0.048 | 0.174 | -0.019 |
|  | (0.026) | (0.258) | (0.029) | (0.053) | (0.500) | (0.056) | (0.021) | (0.222) | (0.025) | (0.029) | (0.277) | (0.031) |
| Age squared | -0.012 | 0.001 | -0.000 | -0.020 | -0.070 | 0.013 | -0.004 | 0.155 | -0.028 | -0.051* | -0.077 | 0.014 |
|  | (0.023) | (0.137) | (0.025) | (0.046) | (0.267) | (0.049) | (0.019) | (0.123) | (0.023) | (0.026) | (0.154) | (0.028) |
| Female | 0.016*** | -0.046* | -0.011* | 0.022** | -0.048 | -0.011 | 0.004 | 0.005 | 0.001 | 0.008 | -0.023 | -0.005 |
|  | (0.006) | (0.028) | (0.006) | (0.009) | (0.043) | (0.010) | (0.004) | (0.019) | (0.004) | (0.006) | (0.026) | (0.006) |
| Education (ref=no schooling) | | |  |  |  |  |  |  |  |  |  |  |
| Primary school or less | -0.001 | -0.014 | 0.004 | -0.003 | -0.006 | 0.002 | 0.000 | -0.001 | 0.000 | 0.001 | 0.001 | -0.000 |
|  | (0.003) | (0.012) | (0.004) | (0.007) | (0.025) | (0.007) | (0.004) | (0.014) | (0.004) | (0.005) | (0.018) | (0.005) |
| Junior high or less | -0.000 | -0.022 | 0.004 | 0.000 | 0.002 | -0.000 | 0.001 | -0.005 | 0.001 | 0.002 | 0.011 | -0.002 |
|  | (0.002) | (0.017) | (0.003) | (0.005) | (0.034) | (0.005) | (0.003) | (0.019) | (0.003) | (0.004) | (0.024) | (0.004) |
| High school or less | -0.000 | -0.011 | -0.002 | 0.001 | -0.009 | -0.002 | -0.001 | -0.001 | -0.000 | -0.002 | 0.007 | 0.001 |
|  | (0.002) | (0.010) | (0.002) | (0.004) | (0.019) | (0.004) | (0.002) | (0.010) | (0.002) | (0.003) | (0.013) | (0.003) |
| Bachelor's degree | -0.004 | -0.005 | -0.004 | 0.008 | -0.013 | -0.009 | -0.002 | -0.000 | -0.000 | -0.007 | 0.008 | 0.005 |
|  | (0.004) | (0.007) | (0.005) | (0.009) | (0.014) | (0.010) | (0.005) | (0.007) | (0.005) | (0.006) | (0.009) | (0.006) |
| Masters or more | 0.001 | -0.001 | 0.000 | 0.001 | 0.003 | -0.001 | 0.000 | 0.001 | -0.000 | 0.001 | 0.002 | -0.001 |
|  | (0.001) | (0.001) | (0.001) | (0.001) | (0.003) | (0.002) | (0.001) | (0.001) | (0.001) | (0.001) | (0.002) | (0.001) |
| Indigenous | 0.000 | -0.003 | -0.001 | -0.000 | -0.004 | -0.001 | -0.000 | -0.000 | -0.000 | 0.000 | -0.000 | -0.000 |
|  | (0.001) | (0.002) | (0.001) | (0.001) | (0.003) | (0.001) | (0.000) | (0.002) | (0.000) | (0.001) | (0.002) | (0.001) |
| Employed | 0.000 | -0.013 | 0.002 | 0.005 | 0.040 | -0.007 | 0.001 | 0.011 | -0.002 | 0.003 | 0.020 | -0.003 |
|  | (0.003) | (0.018) | (0.003) | (0.005) | (0.032) | (0.005) | (0.002) | (0.014) | (0.002) | (0.003) | (0.020) | (0.003) |
| Marital status (ref=single) | | |  |  |  |  |  |  |  |  |  |  |
| Currently married | 0.004* | 0.031* | -0.005* | -0.001 | 0.015 | -0.002 | -0.001 | -0.006 | 0.001 | -0.002 | -0.013 | 0.002 |
|  | (0.003) | (0.017) | (0.003) | (0.005) | (0.036) | (0.005) | (0.002) | (0.012) | (0.002) | (0.002) | (0.015) | (0.002) |
| Formerly married | -0.002 | -0.001 | 0.000 | -0.002 | 0.006 | -0.002 | -0.001 | -0.001 | 0.000 | -0.001 | 0.002 | -0.001 |
|  | (0.002) | (0.005) | (0.002) | (0.003) | (0.008) | (0.003) | (0.001) | (0.003) | (0.001) | (0.001) | (0.004) | (0.001) |
| Lives with other relative | -0.001** | 0.001* | 0.001 | -0.003*** | 0.002** | 0.002** | -0.001** | 0.001 | 0.001 | -0.001** | 0.001* | 0.001* |
|  | (0.001) | (0.001) | (0.001) | (0.001) | (0.001) | (0.001) | (0.000) | (0.000) | (0.000) | (0.000) | (0.000) | (0.000) |
| Number of children in house | -0.001 | -0.017** | 0.002* | -0.003 | -0.006 | 0.001 | 0.000 | 0.004 | -0.000 | 0.001 | 0.006 | -0.001 |
|  | (0.001) | (0.008) | (0.001) | (0.002) | (0.018) | (0.002) | (0.001) | (0.011) | (0.001) | (0.001) | (0.012) | (0.001) |
| Wealth index | 0.002 | 0.001 | -0.001 | 0.005** | 0.005* | -0.004* | 0.001 | 0.001 | -0.001 | 0.000 | -0.001 | 0.001 |
|  | (0.001) | (0.002) | (0.001) | (0.002) | (0.003) | (0.002) | (0.001) | (0.001) | (0.001) | (0.001) | (0.002) | (0.001) |
| Migrated to both | -0.000 | -0.000 | 0.000 | -0.003 | -0.003 | 0.003 | 0.006 | 0.006 | -0.006 | 0.003 | 0.003 | -0.003 |
|  | (0.014) | (0.014) | (0.014) | (0.026) | (0.026) | (0.026) | (0.015) | (0.015) | (0.015) | (0.017) | (0.017) | (0.017) |
| Constant |  | 0.040 |  |  | -0.096 |  |  |  |  |  |  |  |
|  |  | (0.122) |  |  | (0.251) |  |  |  |  |  |  |  |
| N | 15,699 | | | 15,693 | | | 15,746 | | | 15,737 | | |
| Notes: * p<0.10 ** p<0.05 *** p<0.01 Heteroskedasticity-robust standard errors in parentheses. All models are estimated separately using a Oaxaca-Blinder decomposition and probability weights.  Source: EDER 2017; ENH 2017 | | | | | | | | | | | | |

| Table A5: Full Oaxaca-Blinder Decomposition Results for Migrants within Mexico | | | | | | | | | | | | |
| --- | --- | --- | --- | --- | --- | --- | --- | --- | --- | --- | --- | --- |
|  | (1) | | | (2) | | | (3) | | | (4) | | |
|  | depression | | | anxiety | | | fatigue | | | pain | | |
| Difference due to: | endow | coef | inter | endow | coef | inter | endow | coef | inter | endow | coef | inter |
| Age | -0.005 | -0.115 | 0.007 | -0.022 | -0.309 | 0.019 | 0.018* | 0.081 | -0.005 | 0.019* | 0.067 | -0.004 |
|  | (0.008) | (0.156) | (0.010) | (0.015) | (0.280) | (0.017) | (0.009) | (0.184) | (0.011) | (0.010) | (0.187) | (0.011) |
| Age squared | 0.001 | 0.072 | -0.008 | 0.014 | 0.170 | -0.018 | -0.021** | -0.040 | 0.004 | -0.025** | -0.057 | 0.006 |
|  | (0.007) | (0.082) | (0.009) | (0.014) | (0.151) | (0.016) | (0.009) | (0.102) | (0.011) | (0.010) | (0.105) | (0.011) |
| Female | 0.001 | 0.005 | 0.000 | 0.002* | 0.017 | 0.001 | 0.001 | 0.007 | 0.000 | 0.001 | -0.010 | -0.000 |
|  | (0.001) | (0.017) | (0.001) | (0.001) | (0.028) | (0.001) | (0.001) | (0.017) | (0.001) | (0.001) | (0.017) | (0.001) |
| Education (ref=no schooling) | | |  |  |  |  |  |  |  |  |  |  |
| Primary school or less | -0.000 | -0.005 | -0.000 | -0.000 | 0.007 | 0.000 | -0.000 | 0.007 | 0.000 | -0.000 | 0.004 | 0.000 |
|  | (0.000) | (0.008) | (0.000) | (0.000) | (0.010) | (0.000) | (0.000) | (0.008) | (0.000) | (0.000) | (0.008) | (0.000) |
| Junior high or less | 0.000 | -0.008 | 0.000 | 0.000 | 0.004 | -0.000 | 0.000 | 0.007 | -0.000 | 0.000 | 0.008 | -0.000 |
|  | (0.000) | (0.013) | (0.000) | (0.000) | (0.017) | (0.000) | (0.000) | (0.013) | (0.000) | (0.000) | (0.014) | (0.000) |
| High school or less | 0.000 | -0.002 | 0.000 | 0.000 | 0.005 | -0.000 | 0.000 | 0.010 | -0.000 | 0.000 | 0.011 | -0.000 |
|  | (0.001) | (0.010) | (0.000) | (0.000) | (0.014) | (0.000) | (0.001) | (0.010) | (0.000) | (0.001) | (0.011) | (0.000) |
| Bachelor's degree | -0.000 | -0.001 | -0.000 | 0.000 | -0.003 | -0.000 | -0.000 | 0.008 | 0.000 | -0.000 | 0.010 | 0.000 |
|  | (0.001) | (0.009) | (0.000) | (0.000) | (0.013) | (0.000) | (0.001) | (0.010) | (0.000) | (0.001) | (0.011) | (0.000) |
| Masters or more | 0.001* | -0.001 | 0.000 | -0.000 | -0.000 | 0.000 | 0.001 | 0.001 | -0.001 | 0.001* | 0.002 | -0.001 |
|  | (0.001) | (0.001) | (0.001) | (0.001) | (0.003) | (0.001) | (0.001) | (0.002) | (0.001) | (0.001) | (0.002) | (0.001) |
| Indigenous | 0.000 | -0.002 | 0.000 | 0.001* | 0.000 | -0.000 | 0.000 | -0.001 | 0.000 | -0.000 | 0.001 | -0.000 |
|  | (0.000) | (0.002) | (0.000) | (0.000) | (0.003) | (0.000) | (0.000) | (0.002) | (0.000) | (0.000) | (0.002) | (0.000) |
| Employed | 0.002*** | 0.017 | -0.001 | 0.002 | 0.033** | -0.002* | -0.001 | -0.005 | 0.000 | 0.000 | 0.004 | -0.000 |
|  | (0.001) | (0.011) | (0.001) | (0.001) | (0.016) | (0.001) | (0.001) | (0.010) | (0.001) | (0.001) | (0.010) | (0.001) |
| Marital status (ref=single) | | |  |  |  |  |  |  |  |  |  |  |
| Currently married | 0.002* | 0.026** | -0.003* | -0.002 | 0.007 | -0.001 | -0.001 | -0.014 | 0.001 | -0.001 | -0.006 | 0.001 |
|  | (0.001) | (0.013) | (0.001) | (0.002) | (0.019) | (0.002) | (0.001) | (0.010) | (0.001) | (0.001) | (0.009) | (0.001) |
| Formerly married | -0.001* | 0.000 | -0.000 | -0.003*** | -0.002 | 0.000 | -0.001 | -0.002 | 0.000 | -0.001** | -0.001 | 0.000 |
|  | (0.001) | (0.003) | (0.001) | (0.001) | (0.004) | (0.001) | (0.000) | (0.003) | (0.001) | (0.001) | (0.003) | (0.001) |
| Lives with other relative | -0.000 | 0.000 | 0.000 | -0.001* | 0.001 | 0.000 | -0.000 | 0.000 | 0.000 | -0.000 | -0.000 | -0.000 |
|  | (0.000) | (0.001) | (0.000) | (0.000) | (0.001) | (0.000) | (0.000) | (0.001) | (0.000) | (0.000) | (0.001) | (0.000) |
| Number of children in house | -0.000 | -0.012** | 0.000 | -0.001 | -0.006 | 0.000 | 0.000 | 0.003 | -0.000 | 0.000 | 0.004 | -0.000 |
|  | (0.000) | (0.005) | (0.000) | (0.000) | (0.011) | (0.000) | (0.000) | (0.007) | (0.000) | (0.000) | (0.007) | (0.000) |
| Wealth index | 0.001*** | 0.001 | -0.000 | 0.001* | 0.001 | -0.001 | 0.001 | 0.000 | -0.000 | 0.001 | 0.000 | -0.000 |
|  | (0.000) | (0.001) | (0.000) | (0.001) | (0.001) | (0.001) | (0.000) | (0.001) | (0.000) | (0.000) | (0.001) | (0.000) |
| Migrated to both | 0.000 | 0.000 | -0.000 | 0.005** | 0.005** | -0.005** | 0.004*** | 0.004*** | -0.004*** | 0.002 | 0.002 | -0.002 |
|  | (0.001) | (0.001) | (0.001) | (0.002) | (0.002) | (0.002) | (0.001) | (0.001) | (0.001) | (0.001) | (0.001) | (0.001) |
| Constant |  | 0.020 |  |  | 0.031 |  |  | -0.077 |  |  | -0.057 |  |
|  |  | (0.081) |  |  | (0.139) |  |  | (0.092) |  |  | (0.101) |  |
| N | 23,429 | | | 23,418 | | | 23,493 | | | 23,485 | | |
| Notes: * p<0.10 ** p<0.05 *** p<0.01 Heteroskedasticity-robust standard errors in parentheses. All models are estimated separately using a Oaxaca-Blinder decomposition and probability weights.  Source: EDER 2017; ENH 2017 | | | | | | | | | | | | |

1. Because the regressions are weighted, we must use the weighted averages from the descriptives table. [↑](#footnote-ref-1)
